# Investigating the knowledge, attitude, and practice of nurses on drowning prevention in primary school children

**Published:** 2023-12

**Authors:** Fatemeh Mansouri, Azar Darvishpour, Shiva Mahdavi Fashtami

**Affiliations:** ^*a* Department of Nursing, Zeyinab (P.B.U.H) School of Nursing and Midwifery, Guilan University of Medical Sciences, Rasht, Iran. ^; ^*b* Social Determinants of Health (SDH) Research Center, Guilan University of Medical Sciences, Rasht, Iran. ^; ^*c* Pirouz Hospital, Guilan University of Medical Sciences, Rasht, Iran. ^

## Abstract

**Background:**

There are limited educational materials regarding drowning prevention. Assessing the awareness, attitude, and practice of health workers will help to design an educational package for drowning prevention. The purpose of this study was to assess the knowledge, attitude, and practice of nurses about drowning prevention in primary school children.

**Methods:**

This cross-sectional study was conducted in June 2023 as an online survey among nurses working in the hospitals of East Guilan in the north of Iran. Forty-eight nurses completed the KAP (Knowledge, Attitude, and Practice) questionnaire about drowning prevention for primary school children. Descriptive statistics and Spearman's correlation were used for data analysis using the DigiSurvey online system and SPSS software (version 21).

**Results:**

The majority of subjects (97.91%) were female and in the age group of 36-45 years (66.7%). Half of the participants had one child, 43.8% had two children, and the rest had 3 children. The majority of respondents (60.4%) stated that they do not take their child to the swimming pool for swimming, but 70.8% stated that during the last 12 months, they did recreational activities related to water with their children. The majority (66.66%) stated that their child has never participated in a swimming class. The mean scores of knowledge, attitude, and practice of the subjects regarding the prevention of drowning of primary school children, were 17.60±4.10 (score range: 0-29), 65.56±6.83 (score range: 22-110), and 83.10±62.9 (score range: 25-100), respectively. There was a significant relationship between knowledge and practice (r=.33, p=.02).

**Conclusions:**

The results showed that the knowledge and attitude of most of the nurses about the prevention of drowning in primary school children was at an average level and they had good practice. Despite this, it seems necessary to plan on improving the level of their knowledge and attitude with the help of the relevant organizations and authorities.

**Keywords:**

Drowning, Prevention, Children, Nurse

